# Endolysins from Antarctic *Pseudomonas* Display Lysozyme Activity at Low Temperature

**DOI:** 10.3390/md18110579

**Published:** 2020-11-20

**Authors:** Marco Orlando, Sandra Pucciarelli, Marina Lotti

**Affiliations:** 1 Department of Biotechnology and Biosciences, State University of Milano Bicocca, 20126 Milano, Italy; m.orlando14@campus.unimib.it; 2 School of Biosciences and Veterinary Medicine, University of Camerino, 62032 Camerino, Italy; sandra.pucciarelli@unicam.it

**Keywords:** cold adaptation, cold-active enzyme, endolysin, glycoside hydrolase 19

## Abstract

Organisms specialized to thrive in cold environments (so-called psychrophiles) produce enzymes with the remarkable ability to catalyze chemical reactions at low temperature. Cold activity relies on adaptive changes in the proteins’ sequence and structural organization that result in high conformational flexibility. As a consequence of flexibility, several such enzymes are inherently heat sensitive. Cold-active enzymes are of interest for application in a number of bioprocesses, where cold activity coupled with easy thermal inactivation can be of advantage. We describe the biochemical and functional properties of two glycosyl hydrolases (named LYS177 and LYS188) of family 19 (GH19), identified in the genome of an Antarctic marine *Pseudomonas*. Molecular evolutionary analysis placed them in a group of characterized GH19 endolysins active on lysozyme substrates, such as peptidoglycan. Enzyme activity peaks at about 25–35 °C and 40% residual activity is retained at 5 °C. LYS177 and LYS188 are thermolabile, with Tm of 52 and 45 °C and half-lives of 48 and 12 h at 37 °C, respectively. Bioinformatics analyses suggest that low heat stability may be associated to temperature-driven increases in local flexibility occurring mainly in a specific region of the polypeptide that is predicted to contain hot spots for aggregation.

## 1. Introduction

Adaptation to life in cold environments translates into a variety of molecular changes in the cell structures and in the features of macromolecules of the so-called psychrophilic organisms [1,2]. The body of information built over the years shows a large diversification in adaptation strategies that pinpoints, beside the relevance of the selective pressure exerted by temperature, also the importance of the evolutionary history organisms followed. This is clearly shown by the variety of sequences, structures, and biochemical properties of cold-active enzymes, where cold activity is defined as the ability to retain relevant residual activity up to temperatures close to 0 °C. It is broadly accepted that most cold-active proteins can cope with cold temperatures since they are endowed with either high global or local flexibility of the protein chain [3,4]. Flexibility often results in the decrease of the thermal stability of the whole protein structure or, in case it is restricted to the active site region, in localized heat lability that triggers inactivation at a temperature lower than the temperature of protein unfolding [5,6]. A trade-off between activity in the cold and stability is reported as a feature of cold-active enzymes. Nevertheless, examples of proteins that are both cold-active and thermo-resistant are reported, suggesting that combination of only apparently contrasting properties can arise from the evolutionary pathway followed by specific sequences [7].

Besides the relevance of understanding the molecular and evolutionary grounds of cold adaptation, enzymes from psychrophilic organisms may find application in biocatalysis and other fields of biotechnology [8,9]. Indeed, high specific activity at low temperature is of advantage for energy saving and in processes in which heat-sensitive substrates are used and side reactions should be avoided, such as, for example, in the food industry and in fine chemistry. A further benefit of psychrophilic and heat-labile enzymes relies on the possibility to inactivate them through small temperature increases [8,10].

Organisms from Antarctic seawater are investigated as sources of materials and biomolecules for application in biotechnology and health care [11,12,13,14,15]. In this frame, we studied Antarctic bacteria and unicellular eukaryotes sampled at Terranova Bay to obtain cold-active enzymes and antifreeze proteins [16,17]. This work focused on glycoside hydrolases of family 19 (GH19) according to the classification of CAZy, the database of carbohydrate active enzymes [18]. GH19 enzymes are endo-glycosidases and hydrolyse β-1,4 glycosidic bonds by inverting the anomeric configuration of the C1 [19]. They are classified either as endochitinases (EC: 3.2.1.14) that cleave glycosidic bonds between N-acetyl-glucosamine (NAG) residues within chitin chains, or as lysozymes (EC: 3.2.1.17) cutting peptidoglycans between N-acetylmuramic acid and NAG residues. Besides, a number of papers [20,21] pointed out that some GH19 proteins are coded by single genes or modular multi-domain lytic gene cassettes in bacteriophages or prophages. A few of these enzymes have been biochemically characterized and classified as endolysins with lysozyme-like properties [22,23,24,25,26,27]. Endolysins are produced in the late phases of the bacteriophage’s lytic cycle and attack the peptidoglycan polymer of the host cell wall, thus allowing for the release of the phage progeny. Recently, a revised classification of GH19 proteins was implemented based on all sequences and biochemical data available in public databases and integrated with evolutionary issues, with the aim to ease the annotation of novel GH19 sequences from genomes and metagenomes, thus making the discovery of new GH19 enzymes of interest for application in biotechnology easier (https://gh19ed.biocatnet.de). In particular, endolysins are under investigation as specific antimicrobial candidates towards Gram-positive bacteria in the frame of the quest for new antibacterial drugs to fight drug resistance [28]. Recent developments raised expectations also for a possible use against Gram-negative bacteria [29].

In this study, we report the biochemical features and the evolution of two cold-active and thermosensitive GH19 hydrolases identified in the genome of an Antarctic marine *Pseudomonas* strain [30,31] and address the functional differences between the two by bioinformatics analysis. To the best of our knowledge, this is the first experimental description of cold-active GH19 proteins.

## 2. Results

### 2.1. Mining and Molecular Evolution of Glycoside Hydrolases from the Bacterial Isolate

The source strain *Pseudomonas* Ef1 was isolated from a microbial consortium previously collected at Terra Nova Bay [30,32] and classified on the basis of its 16S rDNA sequence [30].

Scanning of the *Pseudomonas* Ef1 annotated genome [28] led to the identification of two putative glycosyl hydrolase coding sequences named lys177 (531 bp) and lys188 (564 bp). Orthologous genes of both l*ys177* and *lys188* were identified in the genomes of *P. koreensis* D-26*, P. moravensis* BS3668, and *P. koreensis IMBL1* (Table 1). Lys177 and lys188 share 100% identity with the respective orthologues from *P. koreensis* D-26, a thermosensitive strain isolated from South Korean paddies [33]. Identity to *P. moravensis* BS3668 (strain of unknown origin) genes is 91.53% (l*ys177*) and 89.51% (*lys188),* while the identity to sequences from *P. koreensis* IMBL1, a strain isolated from South Africa, is 94.53% (l*ys177)* and 87.27% (*lys188*), respectively. These results suggest a close relationship of *Pseudomonas* Ef1 with *P. koreensis* D-26, supported also by the high identity shared by their 16S rDNA sequences (Table 1). It is worth noticing that *P. fluorescens* 90f12-2 (16S rDNA sequence 99.93% identical to that of *Pseudomonas* Ef1) lacks the orthologous of Lys 177, as other more distantly related *Pseudomonas* strains. This observation hints to the occurrence of independent phage infections for the integration of phage enzymes into the *Pseudomonas* genomes. According to Phaster (PHAge Search Tool Enhanced Release) [34], both the *lys177* and the *lys188* coding sequences are inserted within different and complete prophagic regions of the *Pseudomonas* host.

The closest match of the deduced amino acid sequences in the UniProtKB/Swiss-Prot database is the GH19 Endolysin A from *Mycobacterium* phage D29 (AN O64203), whose catalytic domain is 37% and 36.8% identical to LYS177 and LYS188, respectively. Signal peptides were not identified.

**Figure 1:**
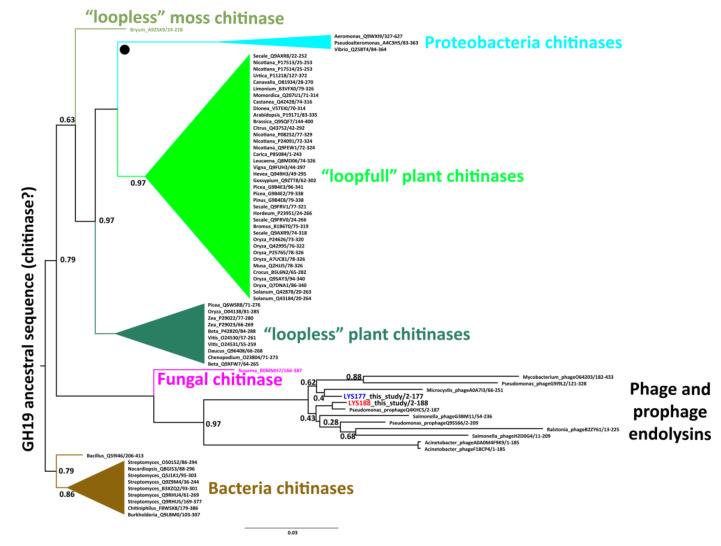
Rooted phylogenetic tree of characterized GH19 proteins. Each tip name represents the organism genus separated by an underscore from the Uniprot AN and by a slash from the sequence start and end position. In the endolysins cluster, the two sequences considered in this study are highlighted in blue and red. Other clusters/tips are coded by different colors and their internal relationships are collapsed for visualization purposes. “●” indicates a hypothetical horizontal gene transfer event. Decimal numbers at internal nodes indicate posterior probabilities only if lower than 1. The branch lengths are proportional to the expected number of substitutions per site.

The evolution of the two sequences within the GH19 family was studied by Bayesian phylogenetic analysis limited to the group of GH19 sequences present in the CAZy and UniProt databases and annotated with biochemical data. We identified five major monophyletic clusters consistent with specific functional and taxonomic groups (Figure 1). Four of them include bacterial chitinases, chitinases from Proteobacteria, and two clusters of plant chitinases differing in the number of substrate binding loops (named with the terms “loopfull” and “loopless” according to [35]). A fifth group contains only phage/prophage endolysins with lysozyme activity. LYS177 and LYS188 nest in this group, suggesting they originated from phage sequences integrated in the *Pseudomonas* genome.

### 2.2. LYS177 and LYS188 are Cold-Active Thermolabile Glycosidases with Lysozyme Activity

The yield of recombinant His-tagged LYS177 and LYS188 (Figure S1), determined after affinity chromatography purification and two buffer exchange steps, was ≈ 2.5 and ≈ 1 mg per 100 mL of culture.

**Figure 2:**
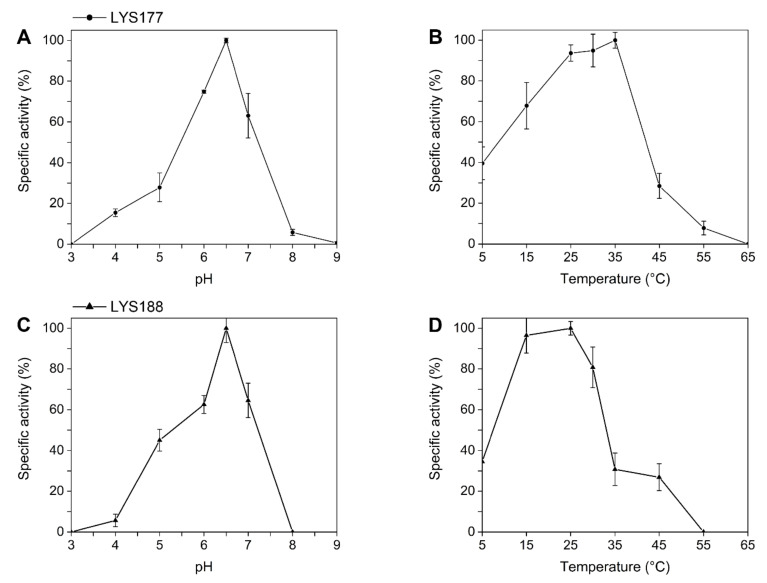
pH and temperature dependence. The effects of pH (**A**,**C**) and of temperature (**B**,**D**) on the specific activity of LYS177 and LYS188 were detected by means of a turbidimetric lysis assay with *M. lisodeikticus* cells. Error bars indicate standard deviations of three independent biological replicates.

Since the GH19 family groups proteins classified either as chitinases or lysozymes ([36] and Figure 1), we assayed recombinant proteins for both activities using the protocols described in the materials and methods section. In the lysozyme-specific assay, the pH_opt_ of LYS177 was 6.5 (Figure 2A) and the highest activity was recorded at 35 °C (Figure 2B). Under optimal reaction conditions, the enzyme-specific activity was 1877 ± 99 U/mg. Moreover, LYS177 retained 40% activity at 5 °C, a hallmark of cold activity. Under the same conditions, the activity of LYS188 was too low to be reliably calculated. For this reason, the buffer concentration was lowered to 50 mM, in order to increase the assay sensitivity [37]. pH_opt_ and residual activity at the low temperature did match those displayed by LYS177, while the temperature supporting the highest activity was close to 25 °C. LYS188 temperature-dependent loss of activity was sharper, with only 30% residual activity at 35 °C. Specific activity under optimal conditions was 142 ± 20 U/mg. Furthermore, endo-chitinase and N-Acetyl-β-glucosaminidase activities, which is the ability to hydrolyze chito-oligosaccharides either at random positions or at the end of the chain, respectively, were assayed using the standard chitinolytic cocktail from *Streptomyces griseus* as the reference. Neither LYS177 nor LYS188 displayed N-acetyl glucosaminidase activity, while traces of endo-chitinase activity were measured (10.5 ± 1.8 and 12.2 ± 2.1, respectively). However, the values determined were over three orders of magnitude lower than that performed by the commercial *Streptomyces* chitinase (22620 ± 150), and similar to those measured for hen egg white lysozyme (8.6 ± 0.7), which is reported to be endowed with a faint activity on chitooligosaccharides [38]. Moreover, LYS177 (but not LYS188) displayed antimicrobial activity on growing cultures of Gram-positive bacteria (Figure S2).

**Figure 3:**
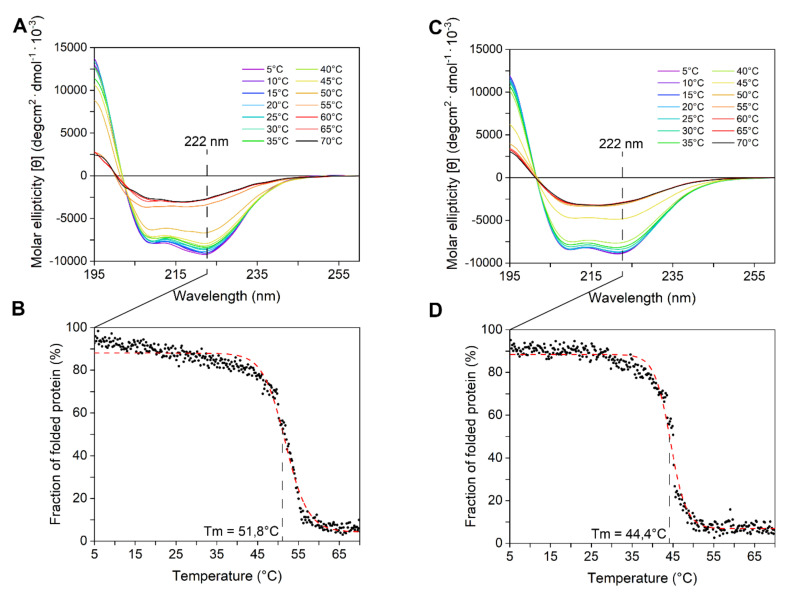
CD spectroscopy analysis. Far-UV CD spectra of LYS177 (**A**) and LYS188 (**C**) recorded at different temperatures. Thermal unfolding of LYS177 (**B**) and LYS188 (**D**) measured at a fixed 222 nm wavelength during heating from 5 to 70 °C. Initial CD signal was taken as 100% for normalization. The Boltzmann fitting was used to estimate T_m_. Data are the average of three independent experiments.

The enzymes’ secondary structure was investigated by circular dichroism performed at 5 °C, to take into account that both proteins are temperature sensitive (Figure 3A,C). CD spectra displayed a bimodal shape, with two local minima at ca. 208 and 222 nm. The observation that the minimum peak at 222 nm was slightly more negative than that at 208 nm suggested (according to [39]) the abundance of α-helices and strong inter-helix interactions. These data are consistent with mainly alpha compact globular domains. The secondary structure content of both enzymes progressively decreased with the temperature. Above 45 °C, the CD signal dropped abruptly because of protein aggregation, which was also detectable as macroscopic precipitates in the cuvette. T_m_, determined at a fixed 222 nm wavelength, was ca. 52 °C for LYS177 and ca. 45 °C for LYS188 (Figure 3B,D).

**Figure 4:**
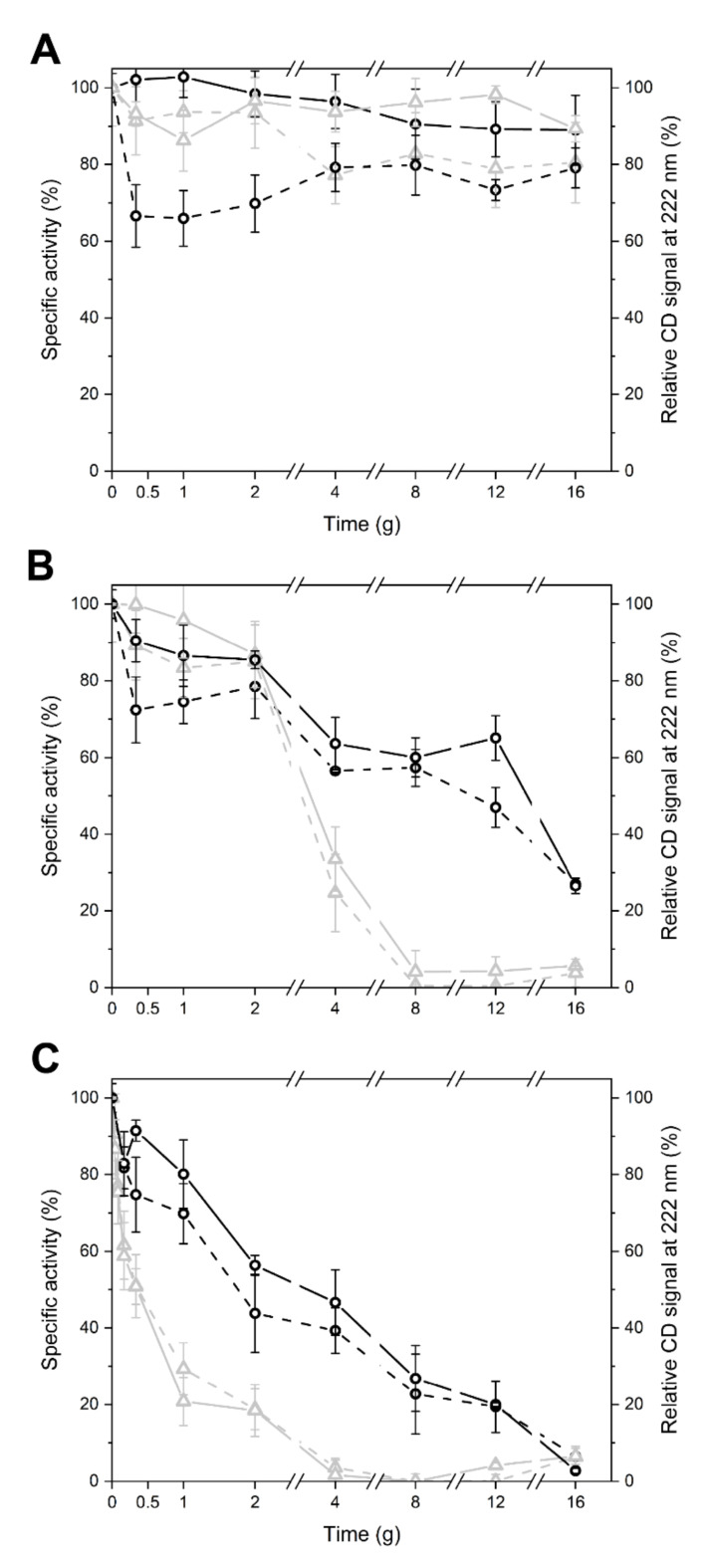
Thermal Stability of LYS177 and LYS188. Relative specific activity (dashed lines) and relative CD signal at 222 nm (continuous lines) of LYS 177 (black circles) and LYS 188 (grey triangles) incubated at 4 (**A**), 25 (**B**), and 37 °C (**C**).

To monitor the effects of temperature over time, the residual activity and residual secondary structure of enzymes, previously exposed to 4, 20, and 37 °C for 30 min up to several days, were measured (Figure 4). In this assay, aimed to assess heat robustness, LYS 177 displayed a peculiar behavior in the first 30 min of the experiment, with a marked drop of activity not accompanied by obvious changes in the secondary structure. This observation is puzzling since under the same conditions, both the activity and structure were unaltered in LYS188, which is less active in absolute values and less stable. In the absence of structural data, we can only hypothesize a fast and/or different rearrangement of a highly flexible region of LYS177 taking place at the very beginning of the experiment (see next section). After this, at 4 °C, the two enzymes were stable for several days, while at 20 and 37 °C, that is under conditions permissive for most proteins but challenging for thermosensitive cold-active enzymes, the activity and secondary structure decreased over time (Figure 4B,C). LYS188 was confirmed to be less robust than LYS177, with half lives of 3 days at 20 °C and 0.5 days at 37 °C vs. 8 and 2 days recorded for LYS177 (Figure 4B,C).

### 2.3. Specific Sequence Signatures May Account for Differences in Heat Stability

**Figure 5:**
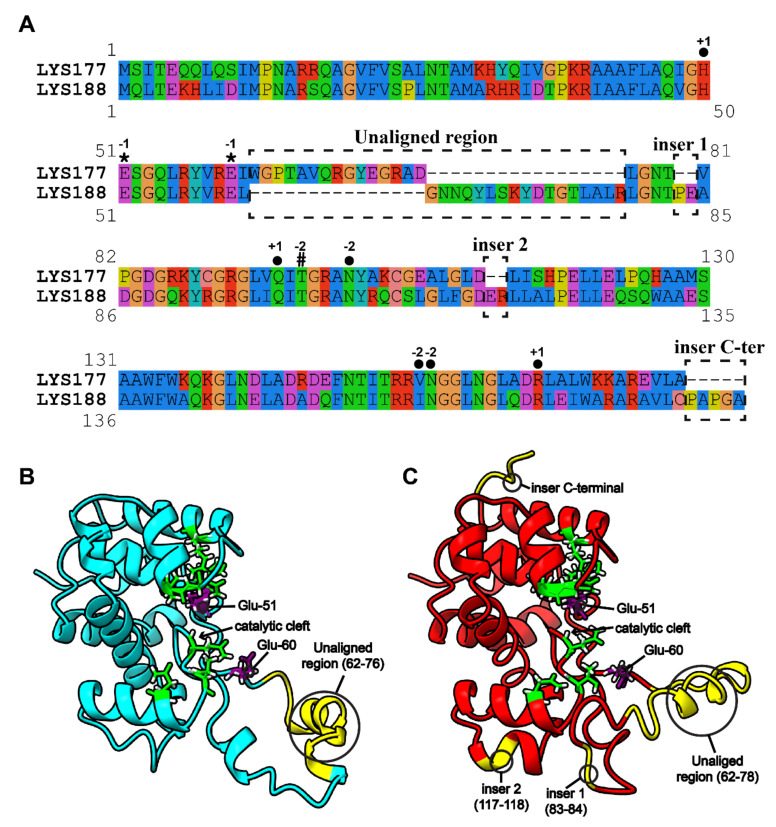
Pairwise alignment and predicted structures. (**A**) Pairwise alignment of LYS177 and LYS188, extracted from the multiple sequence alignment, including close homologues of both enzymes (Figure S3). Annotations of functional relevant sites conserved in the alignment were extracted from the sequences annotated in the GH19 engineering database (accessible at https://gh19ed.biocatnet.de: Sequence ID is 12,365 for LYS177 and 11,988 for LYS188) and marked, as follows, *: catalytic residues; **#**: water coordination residue; ●: substrate binding residues. Numbers indicate the subsite occupied by the sugar moiety predicted to interact with that residue, according the nomenclature reported in [41]. Unaligned/insertion regions are highlighted by dashed boxes. 3-D models of LYS177 (**B**) and LYS188 (**C**) are shown in cyan and red cartoons, respectively. Catalytic glutamate residues are shown as purple sticks. Other residues conserved in the catalytic core are shown as green sticks. Unaligned/insertion regions between close homologues of LYS177 and LYS188 are colored in yellow.

The issue of the different stability towards temperature shown by the two enzymes was addressed by bioinformatics analysis. The first step was the search for specific sequence signatures. To this aim, the recently established GH19 Engineering Database (accessible at https://gh19ed.biocatnet.de; [40]), which contains manually curated annotations and a sequence-based classification of proteins containing a GH19 domain, was used to extract annotations and to download other putative endolysins from the homologous family “*Pseudomonas* prophage like”. In total, 12 sequences from this family were closely related to LYS177 and 82 to LYS188. The resulting multiple alignment is shown in Figure S3. For the sake of simplicity, Figure 5A presents only the LYS177-LYS188 pairwise alignment, yet retaining information and annotations provided by the multiple alignment. LYS sequences share the two catalytic glutamate residues (Glu-51 and Glu-60) and the water coordination residue (Thr-97 for LYS177 and Thr-101 for LYS188), as well as another six residues predicted to be involved in substrate binding from subsite −2 to +1, which are part of the substrate binding “core” conserved in all GH19s (https://gh19ed.biocatnet.de). In the multiple alignment, sequences clustered in two groups, according to the similarity to either LYS177 or LYS188, that differ from each other in a specific region, located right downstream from the catalytic residues and marked in Figure 5 as the “unaligned region”. This region covers amino acids 62–76 of LYS177 and 62–78 of LYS188. Moreover, three different small insertions are present in LYS188, two including a couple of residues (83–84 and 117–118) and the third formed by five C-terminal residues. Positions quoted are highlighted on the 3-D models shown in Figure 5B,C.

**Figure 6:**
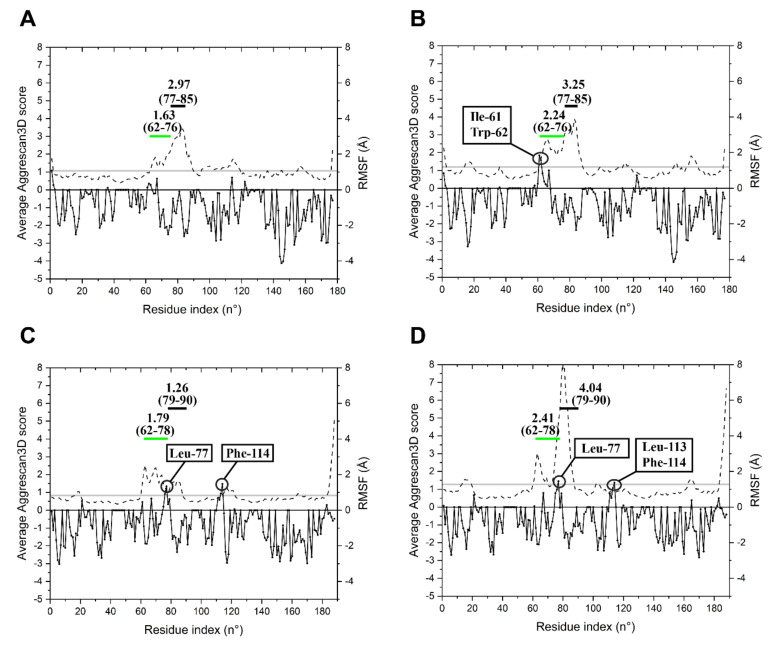
Per residue average Aggrescan3D score and RMSF. MD simulations of LYS177 at 5 (**A**) and 25 °C (**B**) and of LYS188 at 5 (**C**) and 25 °C (**D**). The black line with filled circular symbols indicates per-residue average Aggrescan3D scores; residues with score > 1 are marked. The black dashed line indicates per-residue RMSF. The overall average RMSF is shown as a horizontal grey bar. The green horizontal bar marks the “unaligned region”. The black bar indicates the sequence region immediately downstream. The two numbers in parentheses above each bar show the range of residues involved. The number above each parenthesis is the average RMSF of the amino acid stretch.

Basic features of the enzymes structural dynamics were collected to predict flexibility and aggregation propensity at different temperatures. Corresponding regions in the two models (from 61 to 83 for LYS177 and 61 to 86 from LYS188) were refined by 40 ns MD simulations, while restraining other positions of the structure, as they could not be aligned to the sequence and structure of SPN1S endolysin (Figure S4), which is the closest match (≈39% identity) with known 3-D structure. Molecular dynamics (MD) simulations were performed at 4 and 25 °C. The values of per-residue root mean square fluctuations (RMSFs) and the average Aggrescan3D score (A3DS) were calculated over the MD simulation frames, to be used as predictors of per-residue flexibility and per-residue aggregation propensity, respectively. MD simulations of LYS177 at 4 and 25 °C (Figure 6A,B) suggest that, besides the expected slight increase of the average RMSF with temperature, the “unaligned region” (62–76) is the protein stretch that undergoes the higher increase in flexibility upon heating. Moreover, at 25 °C, Ile-61 and Trp-62 acquire a value A3DS > 1 in the aggregation analysis that predicts a hotspot of aggregation. In LYS188 simulations (Figure 6C,D), the overall RMSF increase at 25 °C is higher than the one predicted for LYS177 as a consequence of a larger RMSF increase predicted at the 79–90 region (corresponding to 77–85 of LYS177), which includes the insertion 1 (83–84, Figure 5). The increase in flexibility of the “unaligned region” (62–78) is similar to that of LYS177 at 25 °C. Contrary to LYS177, in LYS188, a hotspot for aggregation is found at both temperatures just at the end of the “unaligned region” (Leu-77). At 25 °C, this residue and the other residues in close proximity form a predicted aggregation hotspot, which is more flexible than the aggregation hotspot found in LYS177 (Ile-61, Trp-62) at the same temperature. Moreover, other two LYS188 residues have A3DS > 1: Phe-114 at both temperatures and Leu-113 only at 25 °C.

## 3. Discussion

The two GH19 glycoside hydrolases described in this paper are associated to different prophagic regions of the same Antarctic *Pseudomonas* species. Both are endowed with lysozyme activity and are evolutionarily nested in a group of sequences characterized as phage endolysins. Overall, collected data supports the hypothesis they are phage enzymes integrated into the *Pseudomonas* Ef1 genome by independent and temporally separated phage infection events. This hypothesis is supported by the data reported in Table 1, suggesting that lys188 transfer preceded that of lys177, which is present only in the genomes of closely related *Pseudomonas* species. The high sequence identity of l*ys177* and *lys188* genes with *P. koreensis* D-26 sequences pinpoints that both sequences may have integrated in the common ancestor of the two strains before the break-up of the Sino-Korean Craton (which also includes the southern parts of the Korean Peninsula) from core Gondwana. The separation of South Korea from Gondwanaland started by the end of the Ordovician (nearly 400 mya) [42], long before the geographical isolation of the Antarctic continent from Gondwana and the formation of the Polar Front, which is a more recent geological event, dating over 60 mya ago [43].

Based on the GH19 tree topology, we can hypothesize that the common ancestor of GH19 proteins was most likely a chitinolytic enzyme, while lysozyme activity arose later. Our analysis does not allow to infer the type of organism that harbored the common ancestor of all GH19s, because the first bifurcation from the tree root is between proteins mainly from Actinobacteria and those from a composite group including bacterial, plant, phage, and fungal proteins. Nevertheless, data support the hypothesis of a more recent horizontal gene transfer from plants to Proteobacteria, forming a distinct lineage before the diversification of “loopfull” plant chitinases. The evolutionary scenario of the GH19 family presented in this work is different from the most recent family revision [44], as it does not surmise a plant origin and secondary transfer to bacteria. We are aware that, in the lack of a dated tree and a comprehensive sequence sampling, relevant elements are still missing for confirmation of the inferred rooting and horizontal gene transfer events. It may also be underlined that the large average pairwise distance (≈60.1 ± 7.3%) in the primary sequence among different clusters of characterized GH19 sequences emphasizes the poor biochemical information available about this protein family.

Both LYS177 and LYS188 proteins display lysozyme activity and conform to the canonical definition of cold adaptation, since they maintain high specific activity at low temperature. Both of them are temperature sensitive. Simulations suggest that an abrupt increase in flexibility of the “unaligned region” takes place in the heated system and may favor protein aggregation. Moreover, the marked heat sensitivity of LYS188 points to a possible effect of a nearby region, in which a small insertion, shared by all LYS188 close homologs, is present.

Therefore, our results highlight the role of amino acid stretches at the surface of LYS177 and LYS188 in modulating flexibility and likely heat instability of the two proteins, in agreement with the suggestions of previous computationa**l** studies that related protein cold adaptation to increased polypeptide flexibility in surface regions far away from the active site [45]. Such sequence patterns are conserved in some orthologous sequences (GH19 family, >80% sequence identity) from organisms adapted to temperate environments. Moreover, the typical features of cold-active enzymes are shared by some GH19 endolysins from mesophilic phages, i.e., ABgp46 from an *Acinetobacter* phage [27]. Such observations may suggest a lack of selective pressure for maintaining or increasing the thermal stability of viral endolysins. As a matter of fact, endolysins are “one-shot” cell wall-degrading tools for the release of progeny virions [46]. On this ground, the hypothesis might be drawn that low stability could have been adopted as a possible strategy to avoid cell lysis, eventually caused by a long-term persistence of released active endolysins, of other bacterial hosts prior to phage infection.

The “unaligned” region is the more remarkable difference between LYS177 and LYS188. Its plastic nature is further supported by the comparison with other related GH19 endolysins (Figure S6), showing that this region is topologically equivalent to a three-helix module involved in peptidoglycan affinity in the *Salmonella typhimurium* phage endolysin [47]. The absence of relevant substrate binding sites in this region and the structural differences observed in the 3-D models rule out a relevant role in catalysis. Rather, it may be hypothesized a contribution of this stretch of amino acids in enzymes affinity toward different types of peptidoglycan modifications, which would explain the differences in activity of LYS177 and LYS188 on the peptidoglycan substrate. In this conceptual frame, it is tempting to hypothesize a possible role of the “unaligned” region in the co-evolutionary phage–host interactions [48] within the group of GH19 endolysins. 

## 4. Materials and Methods

### 4.1. Identification of the Bacterial Strain and of Glycoside Hydrolases Sequences

*Pseudomonas* Ef1 is a Gram-negative bacterium that was isolated from the microbial consortium previously described as associated to the psychrophilic Antarctic ciliate *Euplotes focardii* [32].

In order to assess the taxonomic position of this strain with respect to other *Pseudomonas* species, the 16S rRNA gene was amplified from the genomic DNA by PCR using bacterial universal degenerate primers 27F (5’-AGAGTTTGATCMTGGCTCAG 3’) and 1492R (5’-TACGGYTACCTTGTTACGACTT 3’), as the forward and reverse primer, respectively. Amplification was in a Biometra Thermal Cycler (Biometra Ltd., Kent, UK) with the following cycling conditions: initial denaturation at 94 °C for 5 min, 30 cycles of 1 min denaturation at 94 °C, annealing at 60 °C for 1 min, and extension at 72 °C for 1 min. A final extension step was at 72 °C for 5 min. Sanger sequencing of the 16S rRNA amplicon was performed by BMR Genomics (Padova, Italy). The rRNA sequence was used as query for a Blastn search on the NCBI data bank (http://blast.ncbi.nlm.nih.gov).

Glycoside hydrolase sequences were identified in the genome deposited in GenBank under the Accession Number (AN) VAUR00000000 by the pipeline available in Prokka 1.12 [49], with the dbCAN database of carbohydrate active enzymes as a reference (http://csbl.bmb.uga.edu/dbCAN). The sequences identified as GH19 were named *lys177* and *lys188* (lys stands for lysozyme and the numbers stand for the amino acid length of the predicted protein sequences). A similar approach was used to annotate the genomes of other *Pseudomonas* species collected from GenBank, to identify orthologues of ly177 and lys188.

SignalP 5 (http://www.cbs.dtu.dk/services/SignalP/; [50]) was used for detecting the presence of signal peptides. The genome of the isolated strain was scan-searched with Phaster [34] to investigate the possible location of genes within prophagic regions.

### 4.2. Enzymes Expression and Purification

The *lys177* and *lys188* gene sequences were codon optimized for expression in *E. coli*, synthesized and cloned into pET-21a expression vector by GenScript USA Inc. (Piscataway, NJ 08854, USA). Both genes are flanked by *Nde*I and *Xho*I restriction sites, and harbor 18 supplemental nucleotides for 6xHis-Tag at their C-terminus. The expression vector was transformed in *E. coli* DH5α (EMD Millipore, Billerica, MA, USA) for amplification and then transferred into *E. coli* BL21[DE3] cells (EMD, Millipore, Billerica, MA, USA) for heterologous production. Transformants were grown overnight at 37 °C in 2 mL of Lysogeny Broth (LB, 10 g/L tryptone, 5 g/L yeast extract, 5 g/L NaCl) and then diluted 1:25 in 50 mL of Zym-5052 medium [51] and incubated overnight at 20 °C. Media contained ampicillin 100 mg/L.

Recombinant His-tagged LYS177 and LYS188 were extracted as described in [52], and purified by immobilized-metal affinity chromatography (IMAC) on Ni/NTA agarose resin (Jena Bioscience, Jena, Germany) at 4 °C after two washing steps at 10 and 20 mM imidazole and elution in 250 mM imidazole, pH 8.0.

Protein concentration was determined by the protein Bradford assay (Bio-Rad, California, USA), using bovine serum albumin as a standard. Samples containing the highest protein concentrations were buffer exchanged twice by gel filtration on a PD10 column (GE Healthcare, Little Chalfont, UK) against 80 mM potassium phosphate buffer, pH 6.5.

Whole-cell extracts, soluble and insoluble protein fractions, and IMAC purified fractions were loaded on 14% acrylamide Tris-Glycine SDS/PAGE with BLUeye Prestained Protein Ladder by GeneDirex Inc. as the standard. After electrophoresis, gels were stained with Coomassie dye (Bio-Rad).

### 4.3. Enzymes Characterization

#### 4.3.1. Lysozyme Activity Assay

Lysozyme activity was measured spectrophotometrically in Euroclone Primo^®^ Multiwell plates 96 (Pero, Italy), by a VICTOR Multilabel Plate Reader (PerkinElmer, Waltham, MA, USA). In total, ≈10 mg/mL cells of the Gram-positive bacterium *Micrococcus lysodeikticus* (Merck KGaA, Darmstadt, Germany) were suspended in 270 µL of 80 mM potassium phosphate buffer, pH 6.5, so that in a single well, A_600_ was between 0.6 and 1. In total, 30 µL (≈2 µg) of enzyme solution were added to the reaction mix and mixed by pipetting. A_600_ was recorded at 10-s intervals up to 10 min. The slope of the linear regression between A_600_ and time (min) was used for the calculation of ∆A_600_/min. Reactions were carried out at 30 °C and the PD10 buffer was used as a blank. In this assay, activity (U) is defined as the amount of enzyme that induces a decrease of 0.001 A_600_ per min, due to *Micrococcus* cell lysis following wall degradation elicited by the enzyme. Specific activity is defined here as U/mg normalized for the reaction volume, according to the formula presented in [53]. HEWL (Hen Egg White Lysozyme, Merck KGaA) was the positive control. Activity was recorded in the 3–9 pH range and in the 5 °C–65 °C (pH 6.5) temperature range. Measures were taken in biological and technical triplicates.

#### 4.3.2. Chitinase Assay

Endo-chitinase activity was measured on the low-molecular-weight fluorimetric substrate 4-methylumbelliferyl β-d-*N*,*N*^I^,*N*^II^-triacetylchitotriose (4-MU chitotrioside) by Merck KGaA. In total, 25 μL of 0.5 mM (0.4 mg/mL) 4-MU chitotrioside solution were added to 20 µL 100 mM K-phosphate (pH 5 or 6.5). Reactions started when 5 µL of enzyme solution (containing either 5 µg or 10 µg enzyme) or the reaction buffer were added to the reaction mixture. After 1 h at 25 °C, 100 µL of sodium carbonate 0.4 M were added to stop the reaction. Fluorescence emitted at 460 nm in 0.1 s by excitation at 355 nm was measured and specific activity determined as:$\frac{\left(F_{enzyme}-F_{buffer}\right)}{10^{3}\cdot time\left(min\right)\cdot mg_{enzyme.}}$. Further, 1 U/mg is the amount of activity per mg of enzyme able to release an amount of 4-MU that will produce a 1000 Approximate Units difference in fluorescence after 1 h in the above reaction mixture.

Chitinolytic activity was measured using the low-molecular-weight chromogenic substrate 4-nitrophenyl *N*-acetyl-β-d-glucosaminide (4-NP chitoside) by Merck KG aA. In total, 25 μL of 4 mg/mL 4-NP chitoside solution were added to 20 µL 100 mM potassium phosphate buffer (pH 5 or 6.5). Reaction started when 5 µL of enzyme solution (containing either 5 µg or 10 µg enzyme) or the reaction buffer were added to the reaction mixture. After 1 h at 25 °C, 100 µL of sodium carbonate 0.4 M were added to stop the reaction. Measuring the A_405_ permitted calculation of the activity based on the molar extinction coefficient of released 4-nitrophenol at pH 10. Further, 1 U/mg is the amount of activity per mg of enzyme that is able to release 1.0 μmol of 4-nitrophenol after 1 h of reaction. Each measure was in triplicate.

#### 4.3.3. Circular Dichroism (CD) Spectroscopy

Measurements of protein samples (6 µM) in 80 mM potassium phosphate buffer pH 6.5 were performed in biological triplicates at 4 °C by a spectropolarimeter J-815 (JASCO Corporation, Easton, PA, USA) in a 1-mm path-length cuvette at variable wavelength in the far-UV range (195–260 nm). Other parameters were: scanning speed 20 nm/min, bandwidth 1 nm, digital integration time per data 2 s, and data pitch 0.2 nm. All spectra were corrected for buffer contribution, smoothed twice by the Means-Movement algorithm, and averaged among different biological replicates.

Thermal denaturation spectra were obtained by measuring the CD signal at 222 nm fixed wavelength when progressively heating the sample from 5 to 70 °C. Measurements were performed with a data pitch of 0.2 °C and a temperature slope of 5 °C/min.

Molar mean ellipticity per residue was calculated according to the formula:

(1) $$\left[\theta \right]=\frac{3300m\cdot \Delta A}{c\cdot n\cdot l}$$

where ∆*A* is the difference in the absorption between circularly polarized right and left light of the protein corrected for blank, *m* is the protein molecular mass in Daltons, *l* is the path length (0.1 cm), *c* is the protein concentration in mg/mL, and *n* is the number of residues [16]. Absolute CD signals were converted to percentage (%) with respect to maximum and minimum values, and the scatterplot with temperature was fitted with a Boltzmann distribution to estimate the thermal denaturation midpoint (T_m_).

#### 4.3.4. Thermal Stability

Relative lysozyme-specific activity and relative CD signal at 222 nm of the two enzyme preparations were determined after incubation at 4, 20, and 37 °C at the pH optimum (pH_opt_) in 80 mM potassium phosphate buffer. Measures were recorded at 25 °C after 4, 8, and 24 h in the first day of incubation and then after day 2 and 4, and every 4 days up to 16 days. If necessary, due to very low stability at 37 °C, the values were also measured after 1, 2, and 12 h. Results were averaged over three biological triplicates.

### 4.4. In Silico Analysis

#### 4.4.1. Phylogenetic Analysis

Multiple alignments and phylogeny analysis were performed with a group of biochemically characterized GH19 selected from CAZy (http://www.cazy.org) and UniProt databases.

A starting approximate alignment was built with the E-ins-I algorithm of Mafft 7.313 [54]. All the accessory domains (not containing the GH19 catalytic domain) were then manually trimmed. A Bio-Neighbour Joining [55] starting tree was generated from this alignment through *Phylogeny.fr* web service (http://www.phylogeny.fr/one_task.cgi?task_type=bionj). These results were refined in a Bayesian analysis by Bali-Phy 3.4 [56]. Six independent Monte Carlo Markov chain analyses were run and stopped after 40,000 cycles, when sampled parameters reached convergence and good mixing according to the manual guidelines (http://www.bali-phy.org/README.html#mixing_and_convergence). In order to eliminate the background noise at the beginning of the run, the first 50% of samples was discarded. Each analysis was performed at default prior parameters, with the LG empirical substitution rate matrix [57] and the rs07 insertion/deletion model [58]. The resulting unrooted tree is the majority consensus from all the samples collected during runs.

The position of the root was inferred with a parsimony-based approach, minimizing the costs of duplication, transfer, and loss events under a defined species phylogeny by RANGER 2 [59], using the previously obtained unrooted phylogeny and a chronogram tree of the species in which each branch represents the evolutionary time. This tree was generated through the “Time Tree of Life” website (http://www.timetree.org/). Three different cost combinations for duplication (D), transfer (T), and loss (L) ([D-T-L]: [2-3-1], [3-3-1], and [2-4-1]) were used to select the optimal position of the root. Each analysis was repeated 100 times. The position of the root was considered reliable if optimal (minimum number of costs) in all attempts.

#### 4.4.2. Alignment and Annotation of Close Homologues of LYS177 and LYS188

The amino acid sequences of LYS177 and LYS188 were Blast searched against the GH19 Engineering Database (accessible at https://gh19ed.biocatnet.de, [40]) to annotate the sequences and extract the sequences of catalytic domains of other GH19s proteins belonging to the same homologous group. Sequences were clustered with CD-HIT [60] at 90% identity to retrieve only closely related orthologous sequences, sharing at least 90% identity with either LYS177 or LYS188. A multiple alignment of these sequences was obtained with the E-ins-I algorithm of Mafft 7.313 [54]. Aligned sites that changed according to a possible recent adaptation specific for LYS177 or LYS188 were inferred by comparing the closely related groups of the two sequences. 

#### 4.4.3. Modelling and Molecular Dynamics (MD) Simulations

Three-dimensional models of LYS177 and LYS188 were built by ITASSER web server [61]. The best models according to the ITASSER scoring scheme were visualized and structurally aligned, with UCSF ChimeraX 0.91 [62], to the closest match available in the PDB database, that is the *Salmonella Typhimurium*-infecting phage SPN1S endolysin (AN 4ok7; [47]).

The protonation states of titratable residues in the LYS177 and LYS188 models were obtained with PDB2PQR [63], estimating titration states with propka 3.1 [64] at pH 6.5. The hydrogenated complex was solvated in a dodecahedron box with a minimum distance of 15 Å from the model and solvated with TIP4P parameterized explicit water molecules according to CHARMM36m forcefield parameters (updated March 2019), downloaded from http://mackerell.umaryland.edu/charmm_ff.shtml#gromacs. K^+^and Cl^-^ ions at a concentration of 80 mM were added to neutralize the charge of the system. An MD simulation was performed in gromacs 2019.6 [65], after prior energy minimization and position-restrained equilibration in the solvent, as outlined by Lindahl for lysozyme in water [66]. The system was equilibrated in NPT conditions at a constant temperature of 4 °C (V-rescale thermostat) and constant pressure of 1 atm (Berendsen barostat), by scaling the center of mass of the reference coordinates with the scaling matrix of the pressure coupling. After equilibration, 40 ns refinement was performed for non-terminal region/s >5 AA in length, if not aligned in their primary sequence between LYS177 and LYS188, or if their modelled structure was not superposed to SPN1S endolysin (Figure S4): All heavy atoms of conserved regions were position restrained with a potential of 1000 kJ mol^−1^ nm^−1^. A simulated annealing protocol was used to heat the refined model equilibrated at 4 °C, up to reach and equilibrate the same system also at 25 °C. Unbiased productive MD simulations with an integration step of 2 fs (covalent bonds between hydrogens and heavy atoms were constrained) were performed at 4 and 25 °C (from respective equilibrated systems after refinement) for 200 ns at least, and continued until convergence of the RMSD values with respect to the starting structure was achieved (Figure S5), saving information on the system every 0.2 ns.

Only the frames sampled in the last 100 ns, after reaching equilibration, were used for calculating per residue RMSF and average aggregation propensity, predicted by applying the Aggrescan3D standalone package [67] to each sampled frame with a 10 Å distance set for the aggregation analysis.

## Figures and Tables

**Table 1:** Pairwise identity (%) of lys177 and lys188 to each other and to their orthologous genes, identified in *Pseudomonas* genomes collected from GenBank. Full-length pairwise identity (%) of 16S rRNA genes is also reported.

Species	Pairwise Identity (%) to lys188	Pairwise Identity (%) to lys177	Pairwise Identity (%) to *Pseudomonas* Ef1 16S	RefSeq Genome Assembly (GenBank AN)
*Pseudomonas* Ef1 (paralogue gene)	61.92 ^a^	61.92 ^a^	100	GCF_007293365.1
*Pseudomonas fluorescens* 90f12-2	93.65	-	99.93	GCF_003732335.1
*Pseudomonas koreensis* D26	100	100	99.87	GCF_001605965.1
*Pseudomonas moravensis* BS3668 ^b^	91.53	89.51	99.87	GCF_900105805.1
*Pseudomonas koreensis* IMBL1	94.53	87.27	99.8	GCF_001856885.1
*Pseudomonas koreensis* BS3658 ^b^	83.6	-	99.8	GCF_900101415.1
*Pseudomonas koreensis* CRS05-R5	83.6	-	99.8	GCF_001654515.1
*Pseudomonas* sp RIT288 ^b^	84.83	-	99.67	GCF_000631985.1
*Pseudomonas granadiensis* LMG 27940	84.13	-	99.54	GCF_900105485.1
*Pseudomonas reinekei* MT1	78.48	-	99.54	GCF_001945365.1
*Pseudomonas koreensis* CI12	82.19	-	98.96	GCF_002003425.1
*Pseudomonas koreensis* P2	68.08	-	98.24	GCF_002177125.1
*Pseudomonas putida* NBRC 14164	-	-	97.98	GCF_000412675.1
*Pseudomonas viridiflava*	-	-	96.54	GCF_900184295.1
*Pseudomonas aeruginosa* PA96	-	-	95.43	GCF_000626655.2

^a^ Pairwise identity between lys177 and lys188 gene, ^b^ More than one lys188 orthologue sequence is present. Only the % identity to the closest sequence is reported; - absent.

## Supplementary Materials

The following are available online at https://www.mdpi.com/1660-3397/18/11/579/s1, Figure S1: SDS/PAGE of recombinant proteins purified by affinity chromatography (IMAC), Figure S2: Antimicrobial plate assay, Figure S3: Multiple alignment of LYS177, LYS188 and close homologues of both enzymes, Figure S4: Structural superposition of LYS177 and LYS188 ITASSER models to the 3D structure of Salmonella Typhimurium-infecting phage SPN1S endolysin (PDB AN 4ok7), Figure S5: Root mean square deviation of the MD simulations during the production run. Figure S6: Multiple alignment of LYS177, LYS188 and other phage homologues.
