# Supplementary material for: Endolysins from Antarctic Pseudomonas Display Lysozyme Activity at Low Temperature
Source: Mar Drugs. 2020 Nov 20;18(11):579. doi: 10.3390/md18110579 (PMC7699920; doi:10.3390/md18110579)
Supplement: Supplementary file 1 [file marinedrugs-18-00579-s001.pdf]

Supplementary Material

Endolysins from Antarctic *Pseudomonas* display lysozyme activity at low temperature

Marco Orlando[1], Sandra Pucciarelli[2], Marina Lotti[1*]

[1] Department of Biotechnology and Biosciences, State University of Milano Bicocca, Milano, Italy

[2] School of Biosciences and Veterinary Medicine, University of Camerino, Camerino (MC), Italy

Correspondence to: Marina Lotti,

Piazza della Scienza 2, 20126 Milano, Italy

Tel: ++39 02 64483527; marina.lotti@unimib.it

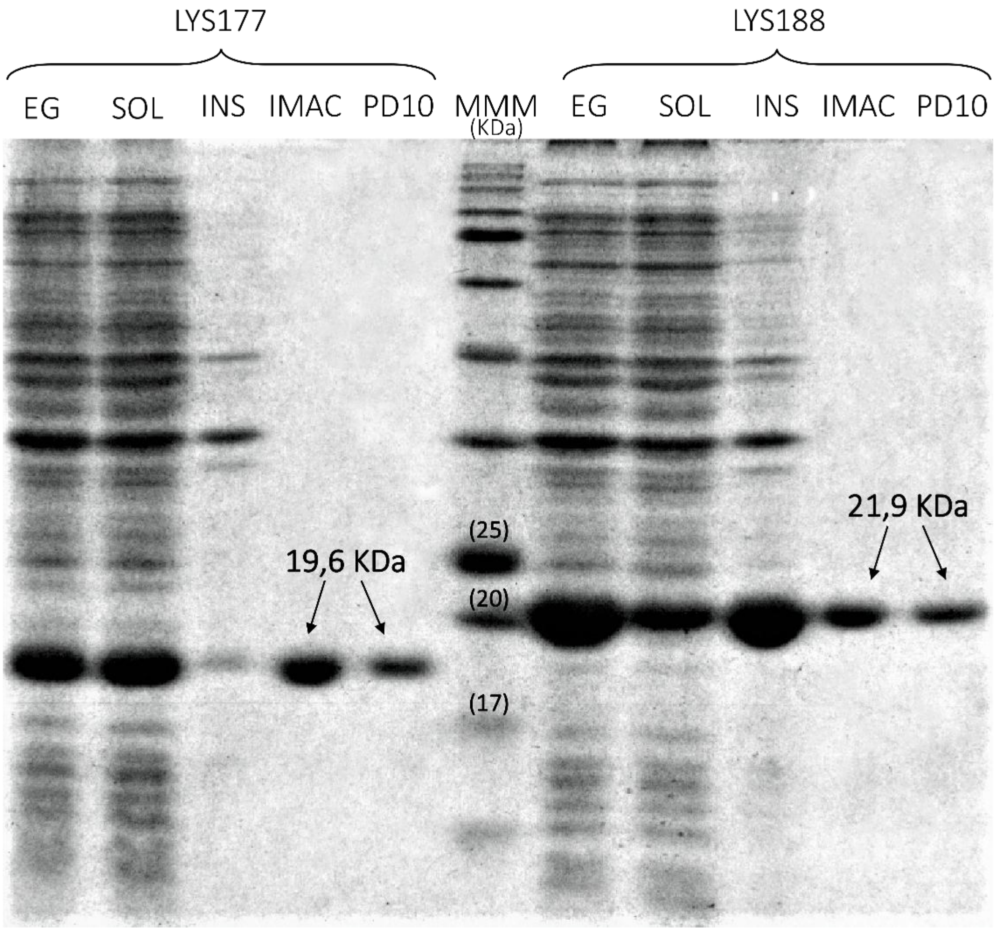

Figure S1. SDS/PAGE of recombinant proteins purified by affinity chromatography (IMAC). For both samples, the first three lanes from the left contain the total (EG), soluble (SOL) and insoluble (INS) fractions obtained from cell lysates after production in Zym-5052 medium (see "Materials and Methods"). ≈ 1,5 µg of proteins was loaded after purification (IMAC) and buffer exchanged by double gel filtration (PD10). MMM: molecular mass marker.

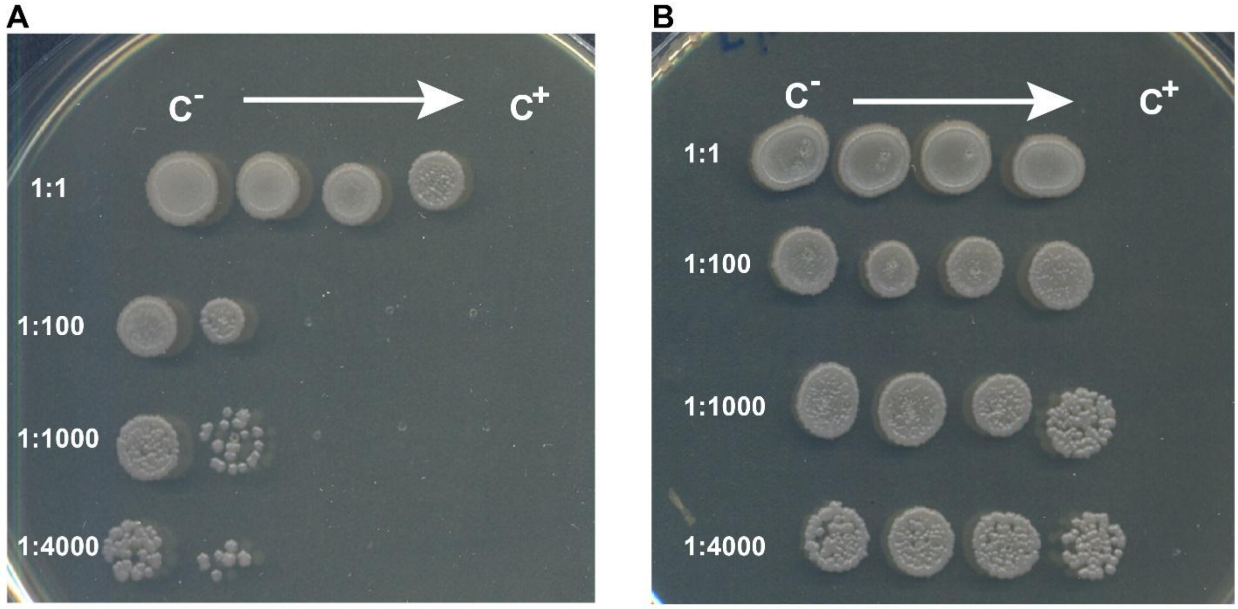

Figure S2. Antimicrobial plate assay. Treatment of different dilutions of exponentially growing *Bacillus subtilis* cultures with LYS177 (**A**) and LYS188 (**B**). The white arrow indicates increasing enzyme amounts: 5, 25 and 50 μg. C: cells treated with buffer only (potassium phosphate 80mM pH 6.5); C+: cells exposed to 5 μg of HEWL. Culture dilution is on the left of each plate row.

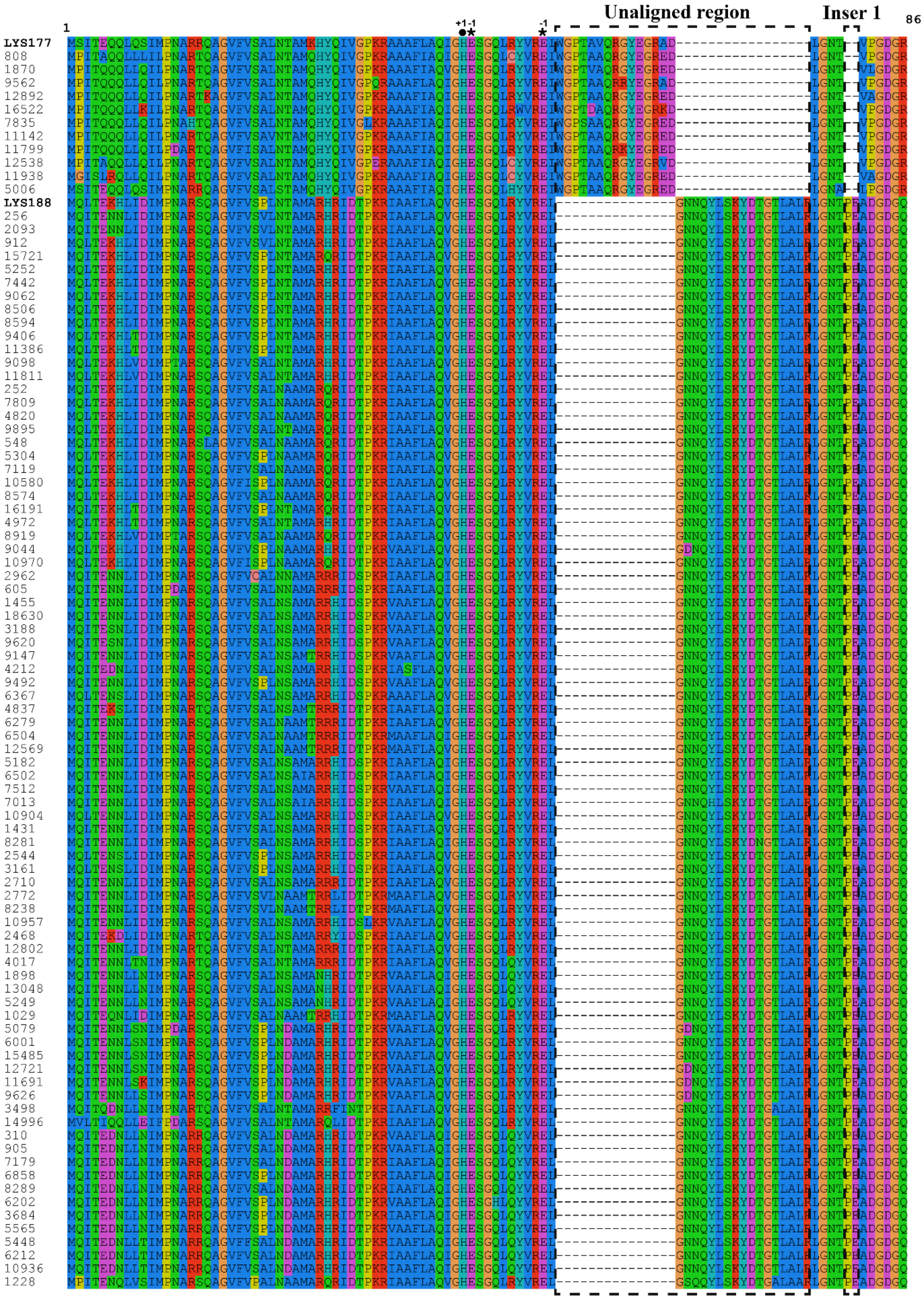

Unaligned region
Inser 1
1
86
LYS177
LYS188

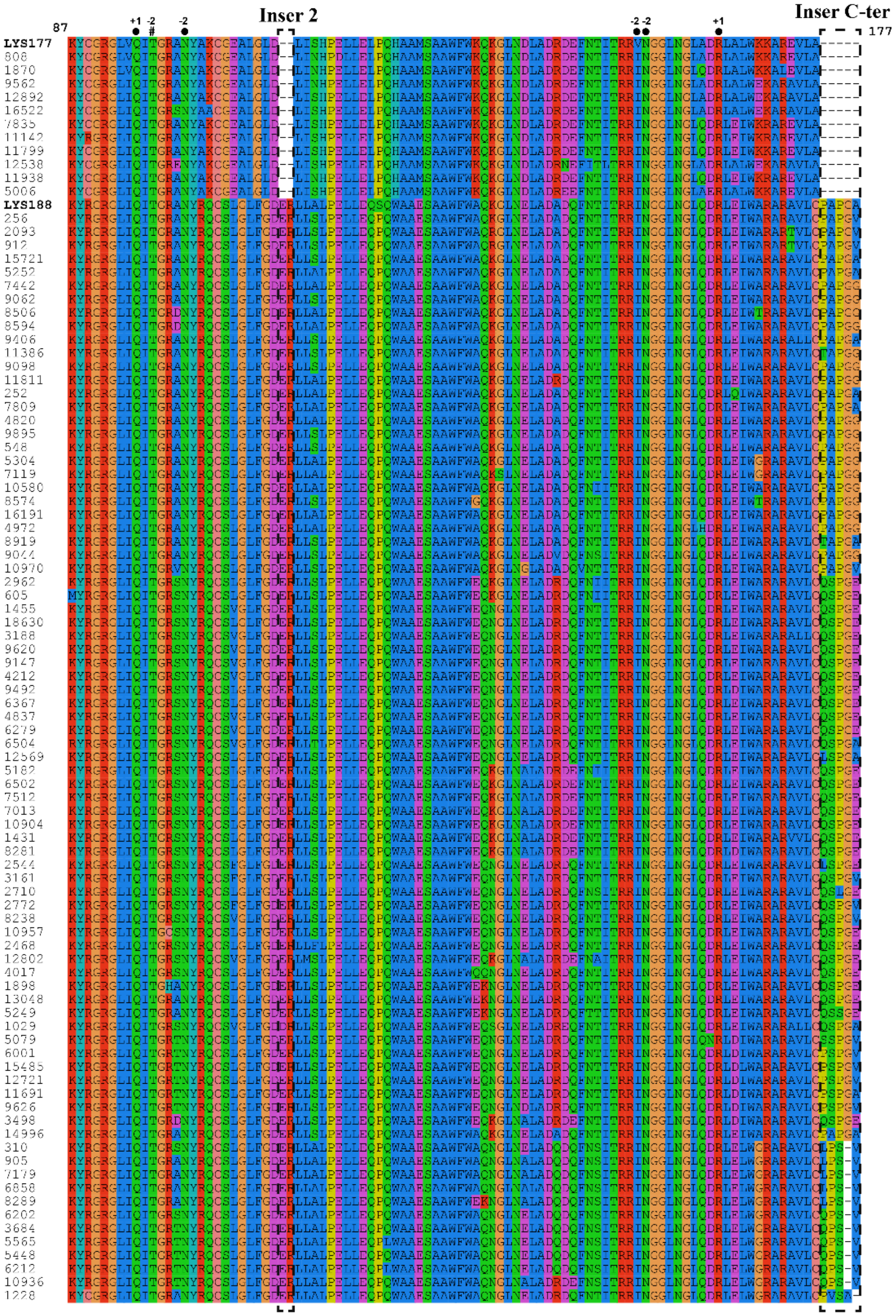

Figure S3. Multiple alignment of LYS177, LYS188 and close homologues of both enzymes. Annotations on functional relevant sites conserved in the alignment were extracted from the GH19 engineering database (GH19ED, accessible at https://gh19ed.biocatnet.de) and reported above each site. Sequence ID is 12,365 for LYS177 and 11,988 for LYS188; other sequences are named according to

their ID in GH19ED. Unaligned/insertion regions are highlighted by dashed boxes. Lower case letters are reported above non conserved sites among the two different groups: close homologues of LYS177 (the first 12 sequences) and LYS188 (the other 83). *: catalytic residues; #: water coordination residue; ●: substrate binding residues. Numbers indicate the subsite occupied by the sugar moiety predicted to interact with that residue, according to [62]. Unaligned/insertion regions are highlighted by dashed boxes. Inser: insertion.

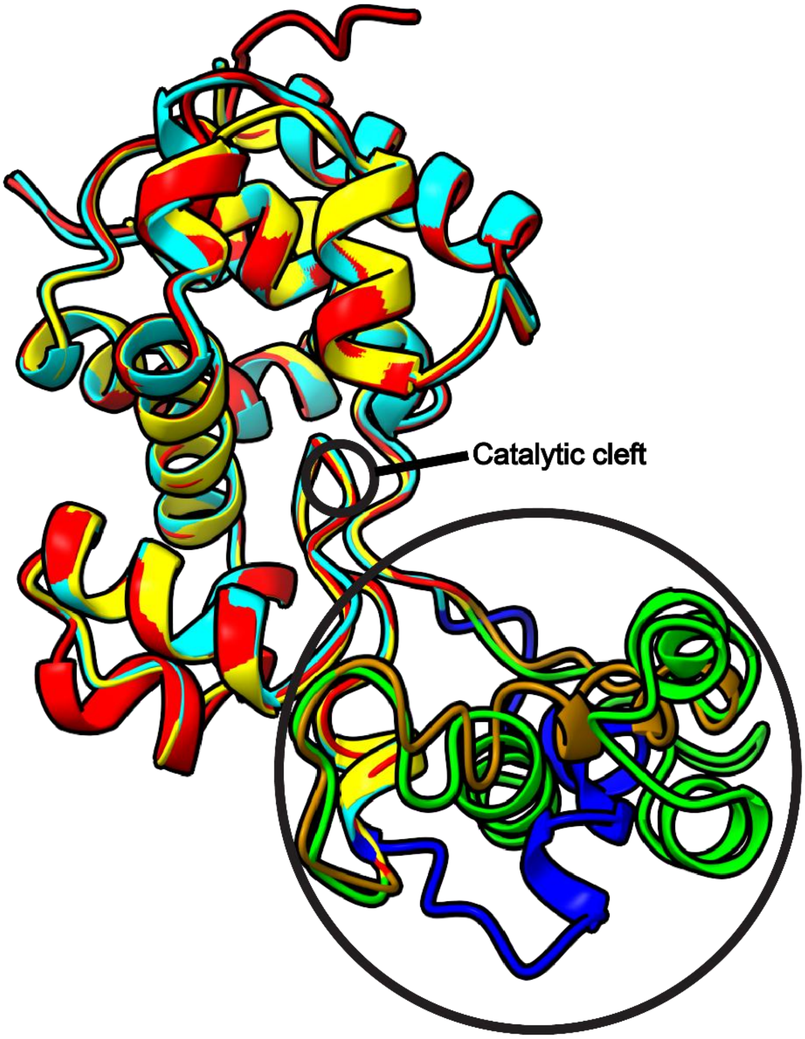

Figure S4. Structural superposition of LYS177 and LYS188 ITASSER models to the 3D structure of *Salmonella Typhimurium*-infecting phage SPN1S endolysin (PDB AN 4ok7). SPN1S endolysin is shown in yellow, LYS177 in cyan and LYS188 in red. A single unaligned region of > 5 AA results from the comparison. This region is coloured in blue in LYS177 (from 61 to 83), dark orange in LYS188 (61 to 86) and green in the SPN1S endolysin (59 to 115). Such amino acids stretches were further refined before MD simulation.

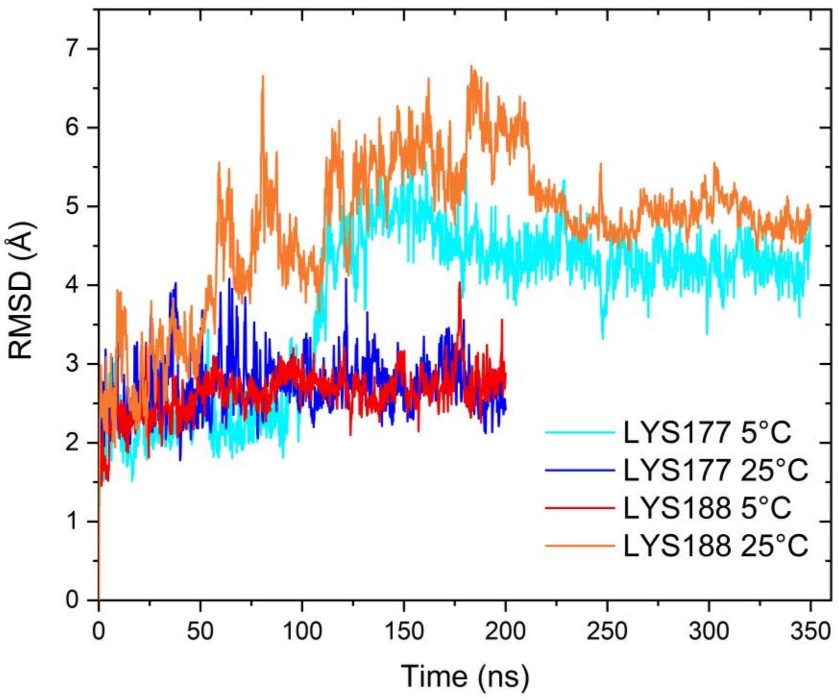

Figure S5. Root mean square deviation of the MD simulations during the production run. The last 100 ns frames of each simulation, collected after stabilization of RMSD values, were used for subsequent analyses.

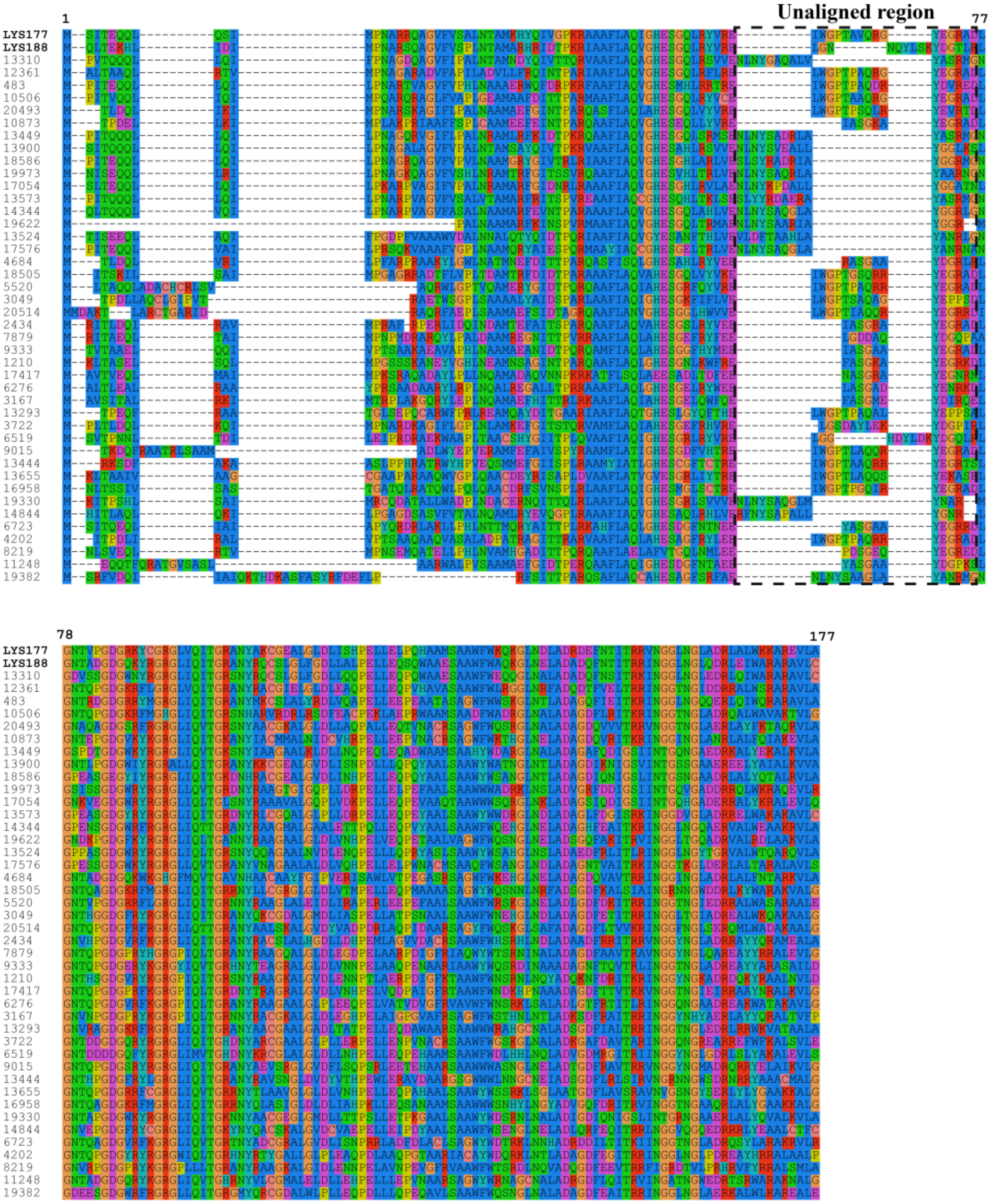

Figure S6. Multiple alignment of LYS177, LYS188 and other phage homologues. 3466 sequences from *Pseudomonas* prophage like homologous group in the GH19ED database (accessible at https://gh19ed.biocatnet.de) were clustered at 60% identity with CD-HIT [60], resulting in 44 centroid sequences; each centroid sequence represents a different portion of the sequence space of the homologous group, avoiding the choice of arbitrary sequences. These centroids sequences, which included LYS177 and LYS188 as representative sequences of their own sequence clusters, were aligned with Mafft 7.313 [54] by using the option 'Leave gappy regions', in order to obtain a global alignment, but avoiding the alignment of locally non-conserved regions.
